# Prevalence and characteristics of avoidant/restrictive food intake disorder in a cohort of young patients in day treatment for eating disorders

**DOI:** 10.1186/s40337-014-0021-3

**Published:** 2014-08-02

**Authors:** Terri A Nicely, Susan Lane-Loney, Emily Masciulli, Christopher S Hollenbeak, Rollyn M Ornstein

**Affiliations:** Penn State College of Medicine, 500 University Drive, 17033 Hershey, PA USA; Division of Adolescent Medicine and Eating Disorders, Penn State Hershey Children’s Hospital, 905 West Governor Road, Suite 250, 17033 Hershey, PA USA; Departments of Surgery and Public Health Sciences, Penn State College of Medicine, 500 University Drive, 17033 Hershey, PA USA

**Keywords:** Avoidant restrictive food intake disorder, Children and adolescents, Day treatment, DSM-5

## Abstract

**Background:**

Avoidant/Restrictive Food Intake Disorder (ARFID) is a “new” diagnosis in the recently published DSM-5, but there is very little literature on patients with ARFID. Our objectives were to determine the prevalence of ARFID in children and adolescents undergoing day treatment for an eating disorder, and to compare ARFID patients to other eating disorder patients in the same cohort.

**Methods:**

A retrospective chart review of 7-17 year olds admitted to a day program for younger patients with eating disorders between 2008 and 2012 was performed. Patients with ARFID were compared to those with anorexia nervosa, bulimia nervosa, and other specified feeding or eating disorder/unspecified feeding or eating disorder with respect to demographics, anthropometrics, clinical symptoms, and psychometric testing, using Chi-square, ANOVA, and post-hoc analysis.

**Results:**

39/173 (22.5%) patients met ARFID criteria. The ARFID group was younger than the non-ARFID group and had a greater proportion of males. Similar degrees of weight loss and malnutrition were found between groups. Patients with ARFID reported greater fears of vomiting and/or choking and food texture issues than those with other eating disorders, as well as greater dependency on nutritional supplements at intake. Children’s Eating Attitudes Test scores were lower for children with than without ARFID. A higher comorbidity of anxiety disorders, pervasive developmental disorder, and learning disorders, and a lower comorbidity of depression, were found in those with ARFID.

**Conclusions:**

This study demonstrates that there are significant demographic and clinical characteristics that differentiate children with ARFID from those with other eating disorders in a day treatment program, and helps substantiate the recognition of ARFID as a distinct eating disorder diagnosis in the DSM-5.

## Background

Historically, children and adolescents have not been easily diagnosed with eating disorders (EDs) based on past versions of the Diagnostic and Statistical Manual of Mental Disorders (DSM), including the 4^th^ edition. In fact, over 50% of these patients met criteria for Eating Disorder Not Otherwise Specified (EDNOS), likely leading to missed diagnoses and difficulty obtaining appropriate and timely treatment [1–3]. With the preparations for publication of the 5^th^ edition of the DSM (DSM-5), the Eating Disorders Work Group was assigned the tasks of improving clinical utility of the diagnostic categories and reducing the frequency of EDNOS. One of the imperatives was to recognize new disorders and eliminate others by exploring the clinical profiles of patients who fell under the heterogeneous EDNOS category. In addition, the DSM-5 as a whole has attempted to take a developmental, or life-span, approach to all disorders.

Feeding Disorder of Infancy or Early Childhood, a diagnosis in the DSM-IV, delineated a persistent eating dysfunction leading to weight loss or failure to gain weight, with the requirement that patients be less than six years of age. This was a non-specific diagnostic category that was rarely used in practice and for which there was insufficient literature [4]. A great number of patients are over six years old at the time of initial ED evaluation, even if some have had symptoms from an early age, and have been necessarily given the diagnosis EDNOS in the past. Feeding Disorder of Infancy or Early Childhood also excluded those children with abnormal eating patterns or nutritionally deficient or limited diets, but who were growing normally secondary to sufficient caloric intake, possibly due to the use of nutritional supplements. The inability of DSM-IV to capture such patients was significant, as they often presented with considerable impairment, both physically and functionally [5].

Clinicians and researchers have long recognized specific types of EDs that fall under the umbrella of EDNOS. The Great Ormond Street (GOS) classification system captured a way to describe these types of patients, and was often utilized by clinicians for descriptive purposes. These criteria were actually found to have a higher inter-rater reliability for younger patients than the DSM-IV [1]. The GOS categories include: Food Avoidant Emotional Disorder (FAED), Selective Eating, and Functional Dysphagia, as well as Anorexia Nervosa (AN) and Bulimia Nervosa (BN).

FAED was first described as a combination of inadequate food intake and emotional disturbance; these young people knew that they were underweight and wanted to be heavier, but found this difficult to achieve [6]. The GOS system further clarified this group, and differentiated their presentation by the absence of weight and shape concerns in the presence of significant food restriction. Somatic complaints were frequent as well as more general psychopathology, e.g. generalized anxiety [4,5].

Selective eating, also known as “picky eating”, is a common problem of childhood, with anywhere between 13 to 22% of children between 3 and 11 years of age being reported to be picky eaters at any given time [7]. While young children are typically thought to “grow out of” their pickiness, studies have shown that between 18 and 40% of the rigidity concerning food persists into adolescence [8–10]. Patients with selective eating are usually not underweight, as they take in adequate calories from preferred foods, but their diets may be lacking in micronutrients. Some selective eaters have sensory concerns related to the taste, smell, color, or texture of foods, which may limit their intake to such a narrow range of acceptable foods that weight loss, or failure to gain appropriate weight, may occur. Studies have shown a higher prevalence of boys with selective eating, as well as a high degree of co-morbid anxiety [11,12].

Functional dysphagia is a fear of swallowing or an inability to eat or swallow food, especially solid or lumpy foods. There is generally a fear of gagging, choking, or vomiting, often subsequent to actual traumatic episodes or witnessed episodes. Sometimes an illogical connection in the child’s mind leads to development of the phobia. Some children present with food refusal specifically out of fears of vomiting, contamination, poisoning, or defecation as well. Many cases of acute food refusal due to specific fears present clinically malnourished and ill, as they often lose weight rapidly. They can easily be mistaken with AN on initial presentation due to the severity of the restriction; however, they are not concerned with weight or shape [4,5].

The DSM-5 has subsumed and expanded Feeding Disorder of Infancy or Early Childhood to capture a greater number of patients who present with avoidant or restrictive eating, but are clearly different from those with AN in that there are no disturbed cognitions about weight and/or shape, or a wish to lose weight. It has been renamed Avoidant/Restrictive Food Intake Disorder (ARFID) and includes those types of patients recognized in the GOS system. Patients with ARFID may present with clinically significant restrictive eating leading to weight loss or lack of weight gain, nutritional deficiencies, reliance on tube feeding or oral nutritional supplements and/or disturbances in psychosocial functioning (see Table 1) [13]. Additionally, they may exhibit similar physical signs and symptoms as patients with AN due to semi-starvation.

**Table 1:** **Diagnostic Criteria for Avoidant/Restrictive Food Intake Disorder**

**WHAT IS ARFID?**	**WHAT ARFID IS NOT**
● A problem with eating or feeding (e.g. seeming disinterest in food or eating; repulsion to certain foods based on their sensory qualities; fears about aversive effects of eating) leading to recurrent inability to take in adequate nutrition and/or energy coupled with one (or more) of the following:	● The eating problems are not due to body image disturbance, and anorexia nervosa or bulimia nervosa cannot be diagnosed instead.
○ Major nutritional deficiency.	● Feeding or eating problems are not the result of scarcity of food or a culturally endorsed tradition.
○ Substantial weight loss (or lack of weight gain).	● The disordered eating is not due to a concomitant medical problem or another psychiatric disorder, so that if the medical or psychiatric disorder is treated, the eating problems resolves.
○ Reliance on nasogastric or gastric tube feeding or oral nutrition supplements.	
○ Impaired psychosocial function.	

Adapted from the Diagnostic and Statistical Manual of Mental Disorders, 5^th^ Edition, American Psychiatric Association, 2013.

Very little has been published on patients with ARFID. Recently, a large multicenter study of children and adolescents presenting as new patients to adolescent medicine ED programs, revealed a 14% prevalence of ARFID, with unique clinical characteristics, including younger age and a greater number of males [14,15]. An 11-year retrospective chart review of adolescent ED patients in Canada reported a 5% prevalence of ARFID [16]. These patients were compared to a matched sample of AN patients, and demonstrated a younger age at presentation, and a higher likelihood of being male. There were specific behaviors and symptoms in the ARFID group, including food avoidance, decreased appetite, abdominal pain, and emetophobia. Both of these studies included all new patients presenting for initial assessments to tertiary care ED programs.

Due to the dearth of literature on ARFID, we sought to determine the prevalence and clinical characteristics of ARFID in young patients admitted to a day treatment program for EDs, and to compare patients with ARFID to those with AN, BN, and Other Specified Feeding or Eating Disorders/Unspecified Feeding or Eating Disorder (OSFED/UFED) in the same cohort.

## Methods

### Participants

A retrospective chart review was conducted on 177 patients admitted to a day program for children and adolescents with EDs between August 4^th^, 2008 and May 1^st^, 2012. This program treats female and male patients, ages 7 to 17 years, with EDs and co-morbid psychopathology. The majority of patients in the program have restrictive EDs, based mostly on the younger average age. However, patients with purging disorders are treated as well. While we treat some patients with sensory features related to food, who may or may not also have an autism spectrum disorder diagnosis, it is important to clarify that patients with longstanding feeding issues and autism are not typically admitted to our program, and are usually managed in the Feeding Disorders Program at our institution.

Initial ED and co-morbid psychiatric diagnoses were made upon admission to the program based on a comprehensive diagnostic psychiatric evaluation, by both a trained child and adolescent psychiatrist and either an experienced clinical psychologist or a licensed social worker/clinical psychiatric specialist, using DSM-IV-TR criteria. Some of the co-morbid diagnoses were based on history conveyed by the parent to the health care provider. DSM-5 ED diagnoses were determined retrospectively and agreed upon together through careful discussion by two of the psychiatric specialists and an adolescent medicine physician, all of whom were personally involved with the cases, using a checklist based on the proposed DSM-5 diagnostic criteria, which were almost identical to the published criteria. Therefore, these diagnoses were not made in a blinded fashion.

Of the 177 eligible subjects, a total of four participants were excluded from the study. Two were excluded for having medical conditions that were retrospectively determined to fully account for their disordered eating behaviors. Two subjects were excluded for having Binge Eating Disorder and composed too small a distinct group for data analysis.

### Measures

#### Demographics, historical and clinical features

Data collected at intake included age, gender, and ethnicity. Historical information included past history of ED and/or other mental health treatment, other medical disorders and consultations by other medical specialists, presence of weight loss, percentage of body weight lost, length of illness, use of nutritional supplements, presence of purging behaviors, excessive exercise, history of food allergies, fears of choking and/or vomiting, and sensory issues related to food. This information was gathered from the initial evaluations by the adolescent medicine physician, the psychiatrist, and the psychologist or clinical social worker.

#### Anthropometrics

Weight and height were measured by trained staff at initial presentation. Gowned weights were obtained on a hospital-grade SECA digital scale and recorded to the nearest tenth of a kg. Heights were measured in bare feet using a fixed stadiometer with a right angle headpiece and recorded to the nearest tenth of a cm. BMI was calculated using the standard formula (kg/m^2^) and the % Median Body Weight (%MBW) was determined based on the 50^th^ percentile BMI-for-age.

#### Psychometric measures

*The Children’s Eating Attitudes Test (ChEAT)* [17] The ChEAT is a 26-item scale assessing attitudes and behaviors associated with food and eating, validated in patients as young as 8 years old, adapted from the original EAT-26 [18]. A score of ≥ 20 is considered clinically significant relative to the normative population. The three subscales reflecting varying types of eating pathology include: Dieting, Bulimia/Food Preoccupation, and Oral Control [18].

*Children’s Depression Inventory (CDI)* [19]. The CDI is a 27-item self-report inventory for assessing depression in children between the ages of 7 and 17 years. The measure yields a Total score (M = 50; SD = 10) and five factors: Negative Mood, Interpersonal Problems, Ineffectiveness, Anhedonia, and Negative Self-Esteem (M = 10; SD = 3).

*Revised Children’s Manifest Anxiety Scale (RCMAS)* [20]. The RCMAS is a 37 item self-report instrument designed to measure anxiety for children and adolescents ages 6 to 17 years. The measure yields a Total Anxiety score based upon 28 items, with 9 items comprising the Lie Scale which is designed to detect responses that are socially desirable. The Total Anxiety Score is expressed as a T-score (M = 50, SD = 10) and there are three factor-based subscales, expressed as scaled scores (M = 10, SD = 3): Physiological Anxiety, Worry/Oversensitivity, and Social Concerns/Concentration.

*The Child Behavior Checklist (CBCL)* [21]. The CBCL provides three global measurements which are expressed as a T- score (M = 50; SD = 10) including Total Score; Internalizing; and Externalizing Scales. In addition, the measure includes 14 Syndrome Scores which reflect clusters of psychiatric symptoms. These scales are also expressed as T-scores (M = 50; SD = 10) and include the following scales: Anxious/Depressed, Withdrawn/Depressed, Somatic Complaints, Social Problems, Thought Problems, Attention Problems, Rule-Breaking Problems, Aggressive Behavior, Affective Problems, Anxiety Problems, Somatic Complaints, ADHD Problems, Oppositional Defiant Problems, and Conduct Problems. It is completed by parents and/or other caregivers.

### Statistical analysis

Analysis included descriptive statistics, chi-square, analysis of variance (ANOVA), and Pearson’s correlation. Bonferroni correction was used to adjust for Type I error, with thresholds set at p < 0.01 for patient characteristics, p < 0.007 for ED symptoms and features, and p < 0.008 for psychiatric co-morbidities. Post-hoc testing to examine between-groups effects was performed with the Hochberg GT2 test. Data were entered and analyzed using SPSS (version17.0, SPSS Inc., Chicago, Illinois).

This study was approved by the Institutional Review Board of the Penn State Hershey Medical Center/College of Medicine.

## Results

### Demographics and anthropometrics

Using the proposed DSM-5 criteria, 39 (22.5%) patients met criteria for ARFID, 93 (53.8%) for AN, 20 (11.6%) for BN, and 21 (12.1%) for OSFED/UFED. Notably, all patients diagnosed with ARFID carried a DSM-IV diagnosis of EDNOS. None were diagnosed with DSM-IV Feeding Disorder of Infancy or Early Childhood, as all were over six years old at intake. Of the 173 participants included, 92% were female with a mean age of 13.5 years (SD = 2.03) (range 7.2 -16.9 years). The cohort was predominantly Caucasian (95%), reflecting the ethnic/racial makeup of the geographic area. There was no significant difference in duration of illness between those patients with ARFID and the other ED groups.

Patients with ARFID were found to be younger than those with other EDs (11.1 years, SD = 1.7 vs. 14.2 years, SD = 1.5; *p* < 0.0001) and to have a greater percentage of males (20.5% vs 4.5%; *p* = 0.008). Of the patients who had lost weight as part of their ED, those with AN lost a greater percentage of their premorbid weight than the other ED groups, including those with ARFID (Table 2). There was a significant difference found in %MBW between those with ARFID and BN, but not between ARFID and AN, or OSFED/UFED (Table 2). While the degree of malnutrition was similar to that of patients with AN, those with ARFID were found to have a greater dependence on nutritional supplements, fears of vomiting and/or choking, and texture/sensory issues pertaining to food (all *p* < 0.0001).

**Table 2:** **Clinical characteristics of patients by eating disorder diagnosis**

	**ARFID**	**AN**	**BN**	**OSFED/UFED**	***p*** **-value**
	**(N = 39)**	**(N = 93)**	**(N = 20)**	**(N = 21)**	
**Patient Characteristics (mean or %)**					
Age (years) (SD)	11.1 (1.7)*	14.0 (1.5)	14.9 (1.1)	14.2 (1.7)	<0.0001
% MBW (SD)	87.1 (13.0)	82.6 (9.2)	108.1 (19.5)*	93.2 (6.8)	<0.0001
% Body Weight Lost (SD)	10.5 (8.4)	18.5 (10.2)*	6.4 (6.5)	14.8 (12.2)	<0.0001
Length of illness (months) (SD)	9.8 (13.2)	8.6 (7.9)	15.9 (11.9)	9.8 (4.9)	N.S.
% Female	79.5	95.7	100	90.5	0.008
% Male	20.5*	4.3	0	9.5	
**Symptoms & Features (%)**					
Enteral Supplement Use	46*	20	0	0	<0.0001
Purge-vomit	0	6	95*	38	<0.0001
Excessive exercise	15*	68	65	52	<0.0001
Food allergy	20	5	10	5	N.S.
Fear of choking or vomiting	44*	1	0	0	<0.0001
Sensory issues	26*	1	0	0	<0.0001
Recent medical specialist consult	46	19	20	33	N.S.
**Psychiatric comorbidities (%)**					
Mood disorder	33*	48	80	76	<0.0001
Anxiety disorder	72*	37	25	14	<0.0001
Autism Spectrum Disorder	13*	0	0	0	0.001
Attention Deficit Disorder	4*	0	1	1	N.S.
Learning Disorder	10*	2	2	0	<0.0001
Cognitive impairment	26*	2	10	0	<0.0001

*Significant finding on post-hoc analysis using Hochberg GT2 test.

AN = Anorexia Nervosa ARFID = Avoidant/Restrictive Food Intake Disorder.

BN = Bulimia Nervosa OSFED/UFED = Other Specified Feeding or Eating Disorder/Unspecified Feeding or Eating Disorder % MBW = % Median Body Weight.

### Psychometric assessment and psychiatric co-morbidities

**Figure 1:**
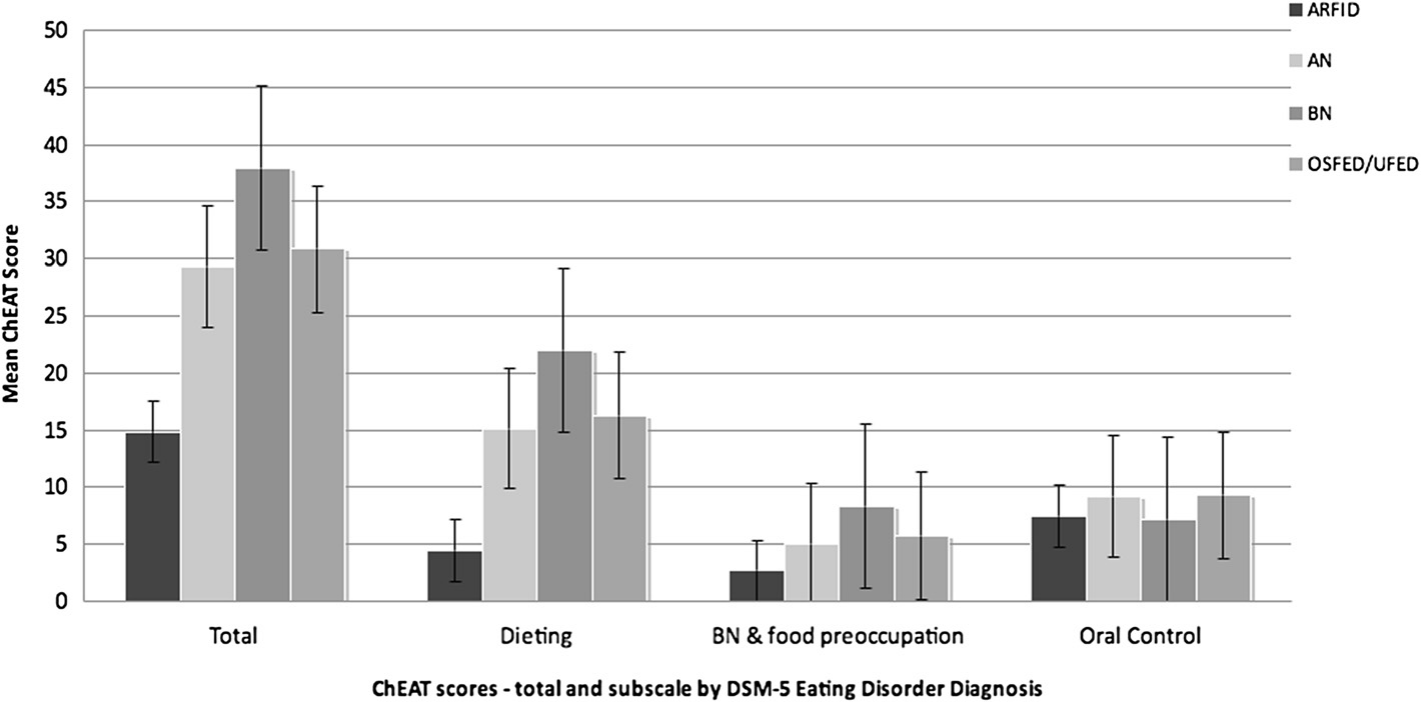
**Total and subscale ChEAT scores by DSM-5 Diagnosis.** ChEAT = Children’s Eating Attitudes Test. All differences between groups significant at p < 0.0001 except Oral Control = N.S.

Patients with ARFID were less likely to report typical ED symptoms, e.g. purging behaviors and excessive exercise, during intake interview (all *p* <0.0001). In addition, they had significantly lower total scores on the ChEAT (14.86, SD = 2.10) than those of the remaining patients overall (27.51, SD = 17.28) (*p* < 0.0001) (Figure 1). Post-hoc analysis revealed significant differences among patients with ARFID and all other groups for the total ChEAT score. While patients with ARFID also had significantly lower scores on both the Dieting and Bulimia Nervosa/Food Preoccupation subscales (*p* < 0.0001), there was no significant difference between groups on the Oral Control subscale. An interesting finding on chart review was that while patients with ARFID did not have true body image distortion, as seen in AN, 21% exhibited body preoccupation with somatic concerns. For example, some children were fixated on fears of physical illness due to issues related to shape/weight, e.g. high cholesterol and/or obesity leading to heart disease, either because of personal experiences with relatives or information in their school curriculum. Others who were chronically underweight due to their feeding and eating disturbance had suffered teasing by their peers because of their low weight, which may have led to body image concerns, although of a different nature than typically seen in AN and BN.

There was a significantly higher comorbidity of anxiety disorders in patients with ARFID (72%) than the other ED groups (31%), as determined by clinician diagnosis (*p* < 0.0001). Furthermore, this was supported by parental report on the CBCL (*p* =0.005). However, there were no significant differences between groups on the total RCMAS score. Autism spectrum disorder (*p* = 0.001), learning disorders (*p* < 0.0001), and cognitive impairment (*p* < 0.0001) were also seen more frequently in the patients with ARFID, based on past history reported at initial assessment (Table 2). On the CBCL, children with ARFID had significantly more social problems (*p* = 0.001) and attention problems (*p* < 0.0001) than those with AN. There was a lower comorbidity of depression diagnosed in children with ARFID (23%) than the other EDs (57%) (*p* < 0.0001), and total CDI scores were lower in this group as well (54.4 vs. 60.0, *p* = 0.05). Additionally, children with ARFID were found to have significantly lower scores on the CDI subscales Negative Mood (*p* =0.02) and Negative Self Esteem (*p* < 0.0001). There were no significant differences between the groups on the Interpersonal Problems, Ineffectiveness, or Anhedonia subscales, however.

A smaller percentage of children with ARFID (35%) sought outpatient psychotherapy before coming to the program, compared to patients in the other ED groups (AN = 60.22%, EDNOS = 75%, BN = 80%; *p* = 0.002). However, there were no differences in the past history of higher levels of psychiatric care, e.g. inpatient, residential, or day treatment. In contrast, more children with ARFID (46.2%) had seen other medical specialists for consultation (e.g. gastroenterology, endocrinology) before coming to program than those with other EDs (26.1%), although this did not reach statistical significance with the Bonferroni correction (*p* = 0.02).

## Discussion

This study adds to the literature on ARFID by comparing a cohort of children and adolescents undergoing day treatment for EDs, including patients with this “new” diagnosis. Notably, almost a quarter of our patients were diagnosed with ARFID, which illustrates the significant prevalence of this disorder amongst children and adolescents requiring an intensive level of ED treatment in a tertiary care setting. This was a higher prevalence than that found in the multicenter studies [14,15], which might be accounted for by the fact that our patients were encountered over four years in a day treatment setting, as opposed to all ED patients presenting for initial evaluation over a one-year period. The prevalence rate was in even starker contrast to the 11-year retrospective review from Canada, where the prevalence was only found to be 5% [16]. There is no mention of age range in that study, only that the patients were adolescent ED patients assessed in a pediatric tertiary care hospital program. Our study included children and adolescents between 7 and 17 years, which may have been a slightly lower range than the Canadian study; this might also justify the higher prevalence of ARFID found in our cohort. Another possible explanation for the discrepancy in prevalence rates across studies is that younger patients with atypical EDs, like ARFID, may be increasingly referred to adolescent medicine ED programs in more recent years, as there has been greater recognition of these presentations as true EDs. The Canadian study reviewed records starting in 2000 and it would be interesting to know whether the prevalence increased annually over the 11 years. In our experience, referrals from primary care providers tend to generate more referrals once they are successfully managed. Lastly, the higher prevalence in our cohort may reflect the fact that many children and adolescents with ARFID present acutely and significantly malnourished, requiring a higher level of care, such as day treatment.

Similar to the multicenter and Canadian studies [15,16], our results demonstrate that there are significant demographic and diagnostic characteristics that differentiate children with ARFID from those with other EDs. First, while female patients remain the majority, there was a higher preponderance of male patients in the ARFID group than in the other ED groups. Children and adolescents with ARFID were more likely to present at a younger age with significant weight loss or failure to gain appropriate weight, were more dependent on oral or enteral nutritional supplementation, and had significantly more fears of choking and/or vomiting, and texture and/or sensitivity issues regarding food. These findings are consistent with those in studies of early-onset EDs [2,22,23], as well as in the recent multicenter study [15], and many are relevant and important features in making the diagnosis of ARFID [24].

Based on DSM-5 criteria, a patient cannot have body image distortion and be diagnosed with ARFID. However, our data revealed that 21% of patients diagnosed with ARFID had body preoccupation with somatic concerns. It is important to reiterate that none of the patients with ARFID had been diagnosed with AN using DSM-IV criteria, which underscores the absence of true body image distortion. During evaluation of a young patient with possible ARFID versus AN, it is critical to probe about body concerns that need to be distinguished from body image distortion. For example, if a patient has worries about becoming fat, this may have something to do with events in the family’s medical history, e.g. an overweight parent or grandparent with a recent myocardial infarction or diabetes diagnosis. Children and adolescents are often privy to this information, but may make illogical associations based on their cognitive developmental stage. This knowledge may then trigger restrictive eating behaviors. Thorough history-taking can often elicit this information.

As has been documented in other studies of patients with acute food avoidance without weight/shape concerns [2,15,22,25,26], there were no significant differences in our study between % MBW in patients with ARFID and AN; however, patients with AN lost a significantly greater percentage of their premorbid body weight. This may be explained by the fact that our patients with ARFID, notably those with the acute food refusal seen in functional dysphagia, may have presented sooner after the onset of illness than those with AN. The data may not fully bear this out due to the heterogeneity of the ARFID category (e.g. more chronic selective eaters vs more acute food refusal), which might balance out the length of illness data. Furthermore, young patients may present relatively early in the course of their illness, based on their age alone.

Based on both clinician and parental report, patients with ARFID had significantly more anxiety and less depression than patients with other EDs, which is similar to findings in the large multicenter study on ARFID [15]. However, our study is the first of patients with ARFID to use standardized measures obtained from parents to aid in evaluation. There were no self-reported significant differences found between children with ARFID and those with other EDs on the RCMAS or any of its subscales, which could be due to the generally high comorbidity of anxiety symptoms in EDs. Alternatively, younger patients (those more likely to be diagnosed with ARFID) may have had a harder time filling out the questionnaire than older subjects, perhaps in understanding the questions or acknowledging symptoms of anxiety, due to cognitive developmental stage. It is important to clarify that ARFID is not simply a type of anxiety disorder, as the severity of the eating disturbance exceeds that which might be seen in an anxiety disorder and necessitates further clinical attention (see Table 1) [13].

Other than the use of outpatient psychotherapy, there were no significant differences between the groups in terms of prior mental health treatment, including hospitalizations for EDs or other mental health issues, admissions to day treatment programs, intensive outpatient programs, or residential treatment facilities. It should be taken into consideration, however, that ours is a young, relatively treatment-naïve population, and that the rate of past mental health admissions would be very different when looking at an older population of patients. Additionally, children with ARFID may be more likely seen as medically ill initially, and the early referrals may tend to gravitate toward the medical as opposed to mental health arena, as a trend in our data revealed, although it was not significant.

There were several strengths to this study, including the large sample size and the use of both clinical and standardized psychometric measures for patient assessment. Additionally, the use of multiple informants (patients, parents, and clinicians) adds to the validity of the findings. Furthermore, experienced clinicians completed all assessments and the adolescent medicine physician involved in deciding on the retrospective DSM-5 diagnoses was integrally involved in the efforts leading up to the inclusion of ARFID in the DSM-5. As ARFID is still a relatively “new” diagnosis, there are no formalized assessment tools available yet. However, instruments will likely be developed, capturing the clinical features and diagnostic criteria which will help standardize diagnosis. There are some available resources to help guide the clinician in evaluation [24,27].

However, there are several limitations that deserve mention. The retrospective nature of this study, and the fact that diagnoses were made on DSM-5 criteria that had not yet been formalized by the time of its completion, need to be taken into consideration. However, as previously mentioned, the published DSM-5 criteria were essentially the same as the proposed criteria used for this study. Careful discussion amongst experienced clinicians very familiar with all of the cases was undertaken to decide upon the appropriate DSM-5 diagnosis for each patient; this did not allow for direct assessment of inter-rater reliability. The absence of blinding of the clinicians may have introduced bias to the outcome of the study, possibly leading to a higher prevalence of ARFID than previously seen in other studies. Lastly, our patients were undergoing day treatment, which implies a certain severity of illness, and may limit the generalizability to patients in other settings, or non-clinical populations. Despite these limitations, this study provides support for ARFID as a separate diagnostic category.

## Conclusions

This is the first study to examine patients with the diagnosis of ARFID in a cohort of patients undergoing day treatment and adds to the limited literature available on this new diagnosis. The inclusion of psychometric measures from both patients and parents has not been documented to date. Children and adolescents with ARFID are clearly distinct from those with other EDs and can now be identified and labeled more specifically and accurately. Ideally, this will enable more timely recognition and access to care. The degree of both physical and psychosocial dysfunction with which these patients present indicates the need for prompt and appropriate treatment. The relatively high prevalence of patients with ARFID in this treatment setting may indicate the need for an intensive level of care for many of these children and adolescents, depending on their initial presentation. Future research on ARFID, with respect to course, prognosis and treatment is warranted.
